# Unsaturated Lipids Change in Olive Tree Drupe and Seed during Fruit Development and in Response to Cold-Stress and Acclimation

**DOI:** 10.3390/ijms17111889

**Published:** 2016-11-12

**Authors:** Simone D’Angeli, Maria Maddalena Altamura

**Affiliations:** Dipartimento di Biologia Ambientale, Sapienza University of Rome, 00185 Roma, Italy; simone.dangeli@uniroma1.it

**Keywords:** cold response, cuticle, fatty acid desaturases, fruit development, linoleic acid, linolenic acid, oil composition, olive tree, seed

## Abstract

The olive tree is a plant of economic value for the oil of its drupe. It is a cultigen complex composed of genotypes with differences in cold-hardiness. About 90% of the oil is stored in oil bodies (OBs) in the drupe during the oleogenic phase. Phenols and lipids contribute to oil quality, but the unsaturated fatty acid (FA) fraction is emerging as the most important for quality, because of the very high content in oleic acid, the presence of ω6-linoleic acid and ω3-linolenic acid, and the very low saturated FA content. Another 10% of oil is produced by the seed. Differences in unsaturated FA-enriched lipids exist among seed coat, endosperm, and embryo. Olive oil quality is also affected by the environmental conditions during fruit growth and genotype peculiarities. Production of linoleic and α-linolenic acids, fruit growth, fruit and leaf responses to low temperatures, including cuticle formation, and cold-acclimation are related processes. The levels of unsaturated FAs are changed by FA-desaturase (FAD) activities, involving the functioning of chloroplasts and endoplasmic reticulum. Cold induces lipid changes during drupe and seed development, affecting FADs, but its effect is related to the genotype capability to acclimate to the cold.

## 1. Introduction

Olive tree (*Olea europaea* L.) is an evergreen woody dicot that has been cultivated in the Mediterranean Basin since ancient times. The plant lacks winter dormancy, and is of economic value for the oil of its fruit, i.e., the drupe. Olive tree ranks sixth in the world’s production of vegetable oils [1], and the areas of the Mediterranean Basin account for more than 90% of the world olive oil production [2]. Interest in olive tree cultivation is progressively extending to countries outside the Mediterranean Basin, e.g., Argentina, Western Africa, Australia, Azerbaijan, China, and Japan [3,4], where the plants have to survive to colder winters [5]. However, many genotypes show low frost-tolerance [6,7] and become less, or non-, productive under unfavourable environmental conditions. Olive tree is a cultigen complex [8], composed of about 2600 genotypes in the Mediterranean regions. The genotypes are usually identified based on the morphological traits of leaves and fruits. This empiric identification is possible because olive tree genotypes retain their genetic characteristics over hundreds of years, with this due to vegetative propagation applied over centuries [2]. It is traditionally known that the oil of some genotypes growing in Mediterranean regions characterized by colder winters is of better quality in comparison with that of genotypes growing under a more temperate climate, e.g., near the sea [9,10].

Some plants tolerate near zero/subzero temperatures by the process known as cold-acclimation, acquired by the exposure to progressively lower, non-freezing, temperatures [11]. In non-cold-acclimated olive tree plants, the cold stress response is characterized by changes intervening at different cell and tissue levels in leaves and twigs [6,12]. The cell membranes are the primary site of cold-induced injury, but, when cold-acclimation is present, it causes the activation of mechanisms protecting membrane fluidity by ensuring the activity of the fluidity-related enzymes [13]. Among these mechanisms there are changes in lipid composition and transient increases in cytosolic calcium levels, both involved in the signal transduction leading to the activation of acclimation-related genes [12,13]. However, how plants decode low temperature message into changes in transcription and lipid composition still remains a widely unresolved question [14], and in woody perennials, in particular. In the latter plants, cold-acclimation generally couples with endo-dormancy and deciduousness, with these processes sharing gene activity, and together causing tree survival in winter [15]. However, there are also trees which are ever-green, lack winter dormancy, but remain able to cold-acclimate [16]. This is the case of the olive tree complex, which includes both cold-acclimating genotypes, e.g., cultivar (cv.) Canino and cv. Frantoio, and cold-sensitive ones, e.g., cv. Moraiolo. In a series of studies with these genotypes, the acquisition or non-acquisition of cold-acclimation was revealed by monitoring the absence and presence, respectively, of cold-induced transient changes of cytosolic calcium levels in protoplasts from both leaves and drupes [5,6,7,12].

## 2. Oil Biogenesis in Olive Tree Drupe and Seed

**Figure 1:**
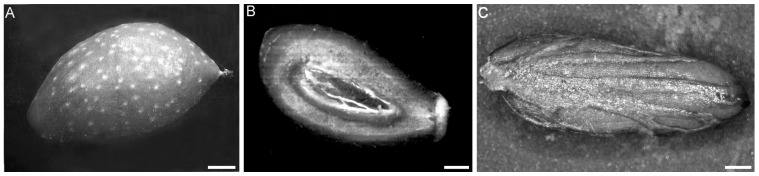
Olive tree drupe and seed at complete development. (**A**) Drupe surface view showing the lenticels (white spots) on the epicarp (cv. Canino, WAF19); (**B**) Longitudinal section of the same drupe showing the presence of one seed in the centre; (**C**) Seed surface view showing the seed coat outer epidermis sculptured by the prominent vascular system (cv. Frantoio, WAF22). WAF, week after flowering. Images under the stereomicroscope. Scale Bars: 3 mm (**A**,**B**), 1 mm (**C**).

The olive tree drupe is formed by an epicarp with stomata at early developmental stages, and lenticels at advanced stages (Figure 1A), a mesocarp in which lipids are stored in oil bodies (OBs), and an endocarp becoming totally lignified at the end of the mesocarp cell expansion growth. Two ovules develop in the ovary, and are both fertilized, however, the fruit endocarp usually encloses only one seed (Figure 1B). The seed is delimited by a seed coat with a well-developed vascular system (Figure 1C), enclosing the endosperm and the embryo [5,17]. In the zones of the Northern hemisphere with Mediterranean climate, fertilization occurs at about the end of May.

The development of the drupe and the deposition of its oil reserves have been described in numerous cultivars. The timing of drupe development and maturation in cv. Canino, cv. Frantoio, and cv. Moraiolo is quite constant over the years, and lasts 18–20 weeks after flowering (WAFs), when the plants of these genotypes are cultivated in pots under the same conditions (standard sandy loam soil, daily irrigation, 41°53′3.5″ latitude north, 12°29′31″ longitude west, 20 m above sea level) [5]. In other cultivars the drupe developmental period may be longer (e.g., in cv. Picual, [18]), and highly dependent on the climate of the year and cultivation area (e.g., in cv. Picual and Arbequina, [9,19]).

**Figure 2:**
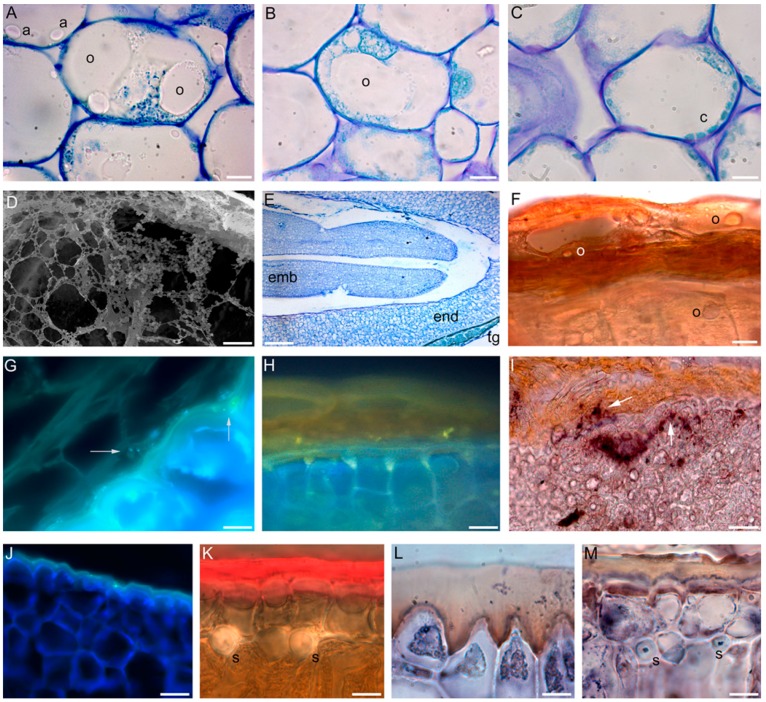
Developmental events related to olive tree oleogenesis and cutinization in the drupe (**A**–**D**,**J**,**L**), in the seed (**E**–**I**), and in the leaves (**K**,**M**), and immunolocalization of osmotin in the same organs (**I**,**L**,**M**). (**A**) Oil body (**o**) formation in the mesocarp cells at WAF7 (cv. Canino). At the same time starch deposition in the amyloplasts (**a**) is still evident; (**B**) Large OB in the cell centre, and small OBs around in a mesocarp cell at WAF9 (cv. Frantoio); (**C**) Chloroplasts (**c**) around a very large OB in a mesocarp cell at WAF10 (cv. Moraiolo); (**D**) Detail under electron microscopy of the protoplast of a mesocarp cell enriched in granules, i.e., plastoglobuli exuded from the plastids (WAF14, cv. Canino); (**E**) Detail of a seed, showing the seed coat with the external epidermis, i.e., tegument (**tg**), already differentiated, the endosperm, and the embryo at the full cotyledonary stage (WAF14, cv. Frantoio); (**F**) Detail of the seed coat and the outermost endosperm containing OBs (**o**) (WAF14, cv. Frantoio); (**G**,**H**) Details of the inner seed coat and outermost endosperm showing spots with cutin autofluorescent signal (**G**, arrow), and cutinized outermost endosperm external walls (**H**) at WAF22; (**I**) Presence of the osmotin signal in some cells of the seed coat internal epidermis (arrow), and in the outer endosperm layers (arrow) (WAF22, cv. Frantoio); (**J**,**K**) Very thick cuticle in the external cell walls of the drupe epicarp (**J**) and the adaxial leaf epidermis (**K**) (WAF19, cv. Canino); and (**L**,**M**) Osmotin immunolocalization signal (intense brown colour) in the inner part of the highly-cutinized external cell walls of the drupe epicarp (**L**), and of the adaxial leaf epidermis (**M**) (WAF19, cv. Canino). Bright-field images after toulidine blue staining (**A**–**C**,**E**), Sudan IV staining (**F**,**K**); and osmotin immuno-labelling (**I**,**L**,**M**); (**G**,**J**) autofluorescence images, (**H**) epifluorescence image after Berberine-HCl staining. Procedures and staining details in [5] (**A**–**D**), [17] (**E**–**J**), and [7] (**K**–**M**). (**a**) amiloplasts, (**c**) chloroplasts, (**emb**) embryo, (**end**) endosperm, (**o**) oil bodies, (**s**) foliar sclereids, (**tg**) seed coat tegument. Histological cross-sections (**A**–**C**,**F**–**M**), longitudinal section (**E**); scanning electron microscopy section (**D**). Scale bars = 10 μm (**A**–**C**,**F**–**M**), bar 3 μm (**D**), and bar 300 μm (**E**).

In Canino, Frantoio, and Moraiolo, under the growth/environmental conditions described above [5], the first developmental stage lasts almost three WAFs, and is characterized by drupe growth through cell division, and by endocarp and seed coat differentiation. The second stage lasts up to WAF6, and is characterized by epicarp and mesocarp growth by cell expansion. The photosynthetic activity is high in this period, and leads to carbohydrate accumulation in the form of starch reserves in the amyloplasts (Figure 2A). During this phase, the vascular system becomes fully differentiated in the mesocarp, and the lignification of the endocarp is completed. In cultivars with a longer fruit developmental period, e.g., cv. Picual, the lignification of the endocarp, the complete differentiation of seed and mesocarp tissues, together with the start of oil accumulation in both tissues, take place later, i.e., at 10–12 WAF [18]. These differences among cultivars are related to a genotype peculiar time course of differentiation, but are mainly dependent on the environmental and cultural conditions, and by a possible alternate fruiting, governed by both plant ageing and external factors [20,21]. However, it is also to be kept in mind that there is a general absence of studies in which the cyto-histological approach is integrated with analytical chemistry techniques.

In Canino, Frantoio, and Moraiolo, the cold responsiveness of the mesocarp cells is high in the second stage, i.e., up to WAF6, independently of the genotype [5]. Lipid storing in the mesocarp cells begins at WAF7 (Figure 2A), when the third stage begins. Oil droplets in the cytoplasm of the mesocarp cells are initially small and about 2–4 per cell (WAF7), but rapidly increase in number (WAF8), and begin to coalesce into oil bodies (OBs). The oil droplets exhibit the remarkable characteristic of lacking oleosins [18], with this allowing coalescence, and being the reason of the easy oil extraction from the mesocarp. At WAF9, about 40% of the mesocarp cell volume is occupied by a large oil droplet, but there are also a lot of minor droplets near the chloroplasts (Figure 2B). At WAF10, i.e., at the end of the stage, part of the chloroplasts turn into chromoplasts, and both the organelle types become enriched in plastoglobuli, and are located around a very large and central OB (Figure 2C). The lipid content becomes many-fold higher than at WAF6, whereas the carbohydrate content does not change. At this time, cold responsiveness decreases [5]. Oil accumulation, i.e., oleogenesis, continues during the fourth stage, lasting 8–10 WAFs, and ceases at the onset of the change in colour of the epicarp, when an intense anthocyanin deposition occurs in the vacuoles of the three cultivars. The cytoplasm of the mesocarp cells becomes granulated (Figure 2D), because enriched in plastoglobuli exuding from the plastids. At the end of oleogenesis, chloroplasts senescence, and the OB occupies about 80% of the mesocarp cell volume [5]. In cultivars with a longer period of mesocarp growth, e.g., Picual, oil accumulation also takes place after the change in colour of the epicarp, although at a lower rate than during the preceding stage [18]. Taken together, results from various cultivars show that there are two possibilities for the end of oil accumulation in the drupe of the olive tree, i.e., at the end of the change in colour of the epicarp [20], and at its beginning [22]. As shown before, the latter is the case for Canino, Moraiolo, and Frantoio [5]. In the latter genotypes, cell viability is reduced to about 20% in the epi-mesocarp at the end of oleogenesis. The cold sensitivity also decreases, but in a genotype-dependent manner. Around WAF19, cold acclimation is acquired by the drupes of the genotypes capable of cold acclimation in the leaves, e.g., Canino, whereas cold sensitivity persists in the drupes of genotypes maintaining cold sensitivity, e.g., Moraiolo [7].

During the following post-maturation phase, i.e., the ripening stage, the mesocarp cell softening and the gradual loss of chlorophyll and carotenoids occur, but with strong differences among the genotypes [23]. The differences in drupe cold-responsiveness remain among the genotypes, however, OB size is constant [5,7].

About 90% of the oil of the drupe is produced by the mesocarp, and accumulates in the triacylglycerols (TAGs) of its OBs [24], however another 10% is produced by the seed, which develops in parallel with the drupe, and also contains lipid reserves, accumulating in the TAGs of the OBs. A histological study in cv. Frantoio [17] has shown that the OBs are present in the seed coat, endosperm, and embryo (Figure 2E,F). In contrast with the OBs of the mesocarp, those of the seed contain oleosins in all the investigated genotypes [25], and the fatty acid (FA) profiles of the TAGs in the OBs differ in FA composition between seed and mesocarp. For example, in cv. Arbequina, at the change in colour stage, palmitic acid and linolenic acid contents in the mesocarp are higher (18% and 1%, respectively) than in the seed (9% and not detected, respectively). On the contrary, oleic acid content is lower in the mesocarp (62%) compared to seed (70%) [26], and the same occurs also in cv. Shengeh [27].

The development of the seed has been described in detail by D’Angeli and coworkers [17] for cv. Frantoio. The protoderm lacks cutinisation from the beginning to the end of embryo formation. The embryo shows the full cotyledonary stage at WAF14 (Figure 2E). OBs are conspicuously present at the same WAF, but their presence does not increase further. Additionally, the endosperm contains OBs (Figure 2F), which exhibit a diameter higher than in the embryo [17]. Differently from the embryo, the outermost layer of the endosperm shows a thick cuticle in the external walls (Figure 2F). Cutin is the major component of the cuticles, and prevalently consists of hydroxyl-FAs, at least in part derived by C18-FAs and glycerol-molecules [28]. Cutin deposition increases during the following weeks in the endosperm, at WAF22 reaching a value about 1.4-fold higher than at WAF14. The seed coat is composed of about 10 cell layers excluding the vasculature. Its outer epidermis also exhibits a cuticle on the external cell walls at WAF14. Internally to the outer epidermis there is a parenchyma with chloroplasts. An inner epidermis with cellulosic cell walls completes the seed coat complex. The cells of all the seed coat layers contain OBs at WAF14, and their number and size do not change further. At WAF22, i.e., during the maturation phase of the drupe, the chloroplasts turn into amyloplasts, and inclusions showing the same autofluorescence signal of cutin, appear in the walls of the internal epidermis (Figure 2G), and in the cuticle of the cell walls of the outermost endosperm layer (Figure 2H). The inner epidermis of the seed coat shows features of secretory epithelium, able to extrude cutin-like materials [17].

Olive tree embryos are already cold tolerant at WAF14, which is many weeks before the completion of drupe maturation and its possible cold acclimation. This tolerance is not related to either an early acquisition of cold acclimation or an early acquisition of dormancy, and continues in time [17]. Dormancy is known to be preceded by desiccation tolerance [29]. In the case of olive trees, embryo cold tolerance seems related to a desiccation tolerance involving a modulation of the levels of oleic and linoleic acids, and cutinisation involving the cooperation of other seed compartments, as described later.

## 3. Olive Oil and Fatty Acid Desaturation

The quality of olive oil is directly related to the physiological state of the drupe from which is extracted [9], and the cold response of the drupe is important [5,7]. Phenolic compounds, lipids, tocopherols, and pigments, such as carotenoids and chlorophylls, contribute to determine the oil quality, and its organoleptic properties. The main part of the organoleptic and nutritional properties of olive oil is determined by the metabolites initially present in the fruit. Among these metabolites, unsaturated FA-enriched lipids and tocopherols do not suffer any relevant transformation during oil processing [19]. Another important category of metabolites accumulating in the mesocarp is represented by secoiridoids, a group of monoterpenoids typical of the Oleaceae and other few dicotyledonous families, present in the drupe as phenol-conjugated compounds. Oleuropein is the most important. Other olive phenolics include phenolic alcohols, e.g., hydroxytyrosol and tyrosol [30]. However, the unsaturated fatty acid (FA) fraction of drupe lipids is emerging as the most important for olive oil quality, because the very high content in oleic acid (C18:1), but also the presence of the ω6 linoleic acid (C18:2), and the ω3-linolenic acid (C18:3), and the very low contents in saturated FAs. This renders olive oil appropriate for human consumption, as well as an excellent fat in diets designed to reduce cardio-vascular diseases, and for the treatment of some cancers and arthritis [24,31].

A positive relationship between composition in unsaturated FAs, fruit growth stage, low temperature response and tolerance, up to freezing tolerance by cold acclimation, has been found in specific olive tree genotypes [5,7].

To change the levels of unsaturated FAs is a feature of the cold-responsive species, and is provided mainly by the regulated activity of FA-desaturases (FADs), involved in adjusting membrane fluidity, in particular [32]. For example, *Arabidopsis fad6* mutant, lacking an active chloroplast ω6 FAD, has reduced levels of polyunsaturated FAs in the chloroplast lipids and altered thylakoids under chilling stress, and *Arabidopsis fad2* mutant, deficient in an endoplasmic reticulum- ω6 FAD, shows decreased polyunsaturates in the extrachloroplast membrane lipids, and a long exposure to cold causes plant withering [33]. Moreover, by over-expressing the *Arabidopsis* chloroplast *ω3 FAD7* gene in tobacco, C18:3 FA increases in the leaves, and the seedling becomes chilling-tolerant [34]. In *Arabidopsis*, AtFAD8 is the plastidial ω3-FAD specifically induced by low temperatures [35], and this enzyme exhibits the same role in other plants, e.g., in rice [36].

The FADs responsible for C18:2 and C18:3 acids from oleic acid are known for the olive tree, and involve the functioning of chloroplasts, chromoplasts, and endoplasmic reticulum (ER). The genes coding these FADs have been investigated, and results about their expression summarized in the following paragraphs.

In addition, recent advances about the changes in response to cold that take place during drupe development, and the *FADs* involved, their regulation by cold, and the effect on the unsaturated FA composition during oleogenesis, have been also summarized and put in relation with the genotype capability to acquire or not acquire cold acclimation, not only in the fruit, but also in the leaves.

## 4. *FAD2.1*, *FAD2.2* and *FAD7* Transcription Characterizes Oil Biogenesis during Drupe Development

The FADs utilize complex lipid substrates [37]. The principal ones are phosphotidylcholine in the ER and monogalactosyl-diacylglycerol in the plastids [38]. Two ω6 FADs, i.e., the plastidial FAD6 and the microsomal FAD2, provide the production of linoleic acid from oleic acid in olive trees [38,39,40]. In cv. Koroneiki, both *OeFAD2* and *OeFAD6* are expressed in various organs, i.e., flowers, fruits, and seeds [2,39]. Moreover, two isoforms of FAD2, i.e., OeFAD2.1 and OeFAD2.2, have been identified in cv. Picual [40]. Two ω3 FADs, i.e., the plastidial OeFAD7 and the microsomal OeFAD3, responsible for the production of C18:3 from C18:2, have been also found in the drupes. *OeFAD3* was initially found in cv. Koroneiki [41], and recently a new *OeFAD3* gene, i.e., *OeFAD3B,* has been identified in cv. Picual and Arbequina [26]. A cDNA sequence assigned to *OeFAD7* was initially cloned by Poghosyan and co-workers in 1999 [42], but the gene was completely sequenced only in 2013 by Sabetta and co-workers in cv. Leccino [43]. Recently, a new *OeFAD7* gene, i.e., *OeFAD7-2,* has been characterized in cv. Picual [26]. Interestingly, it has been demonstrated that there is a total similarity between *OeFAD7* and *AtFAD7* [43]. In *Arabidopsis*, AtFAD7 and AtFAD8 are isoforms [35]. The possible existence of an *OeFAD8* gene has been mentioned for the first time by Poghosyan and co-workers in 1999 [42]. The first primer sequences for an *OeFAD8* gene, i.e., CTTCGTCACTTACTTGCACC and CTCTCAGGTAACTCCATTCC, were designed using *AtFAD8,* and used in cv. Frantoio, Moraiolo and Canino [5]. Recently, about the 98% of the olive tree genome has been sequenced in cv. Farga [44], including a gene with 77.5% identity with *AtFAD8*, to which the sequences firstly identified belong.

By a comparative transcriptional analysis, the expression of *FAD* genes has been monitored during oil biogenesis in drupes of Canino, Moraiolo, and Frantoio [5]. In the forming drupe of WAF3, *OeFAD2.1*, *OeFAD2.2*, *OeFAD3*, *OeFAD6*, and *OeFAD7* are present at a very low level [5]. However, the transcript levels of *OeFAD2.1* and *OeFAD7* increase at the end of the third stage (WAF10), whereas those of *OeFAD6* and *OeFAD2.2* remain quite constant [5]. In contrast, *OeFAD3* levels greatly decrease, becoming hardly detectable. In Canino and Frantoio, the highest *OeFAD2.1* and *OeFAD7* levels occur at the onset (WAF11–12) and near the end (WAF17) of the fourth stage, whereas the *FAD* levels in Moraiolo are always much lower than in the other two genotypes, without any prevalent *FAD* after WAF10 [5]. However, independently of the genotype, the *OeFAD2.1* transcripts are always many fold higher than those of *OeFAD2.2*. All of the above-cited *FAD* transcripts are observed only in traces at the completion of the change in colour of the drupe, independently of the genotype [5].

*FAD* gene expression has been also studied during drupe development and ripening in numerous other cultivars, e.g., cv. Koroneiki [39,41,42], cv. Picual and cv. Arbequina [19,26,40], and cv. Mari and cv. Shengeh [27]. Even if the environmental/growth conditions are different in the different studies, collectively results support either a cultivar-dependence or a cultivar–independence of *FAD* gene expression. In accordance with the first possibility, when four cultivars, i.e., Mari, Koroneiki, Shengeh, and Arbequina, have been compared in the same natural environment, the linoleic acid percentage of Shengeh and Arbequina has been remarkably higher than in Mari and Koroneiki. However, the Shengeh mesocarp always showed higher linoleic acid content than Arbequina, and differences in the timing of *OeFAD2-1* and *OeFAD2-2*, in particular, paralleled these changes [27]. By contrast, according to the second possibility, results from Picual and Arbequina, in comparison, show that the content of linolenic acid is very similar in the mesocarp tissue of both cultivars, and is in both genotypes related with low expression levels of *OeFAD3A* and *OeFAD3B,* and high levels of *OeFAD7-1* and *OeFAD7-2* [26].

## 5. Cold Regulation of *FAD2.2* and *FAD7* Expression in the Drupe

The transcriptional analysis conducted on genotypes with differences in cold acclimation at the end of oleogenesis, i.e., the cold-acclimated Canino and the non-acclimated Moraiolo, demonstrates that *FAD* gene expression during the drupe oleogenic phase also changes in response to the environment, e.g., a cold stress [5]. In fact, after exposure at 6 °C for 24 h at WAF10, *OeFAD2.2*, and *OeFAD7* expression profiles increase similarly in the two genotypes, whereas *OeFAD2.1* and *OeFAD6* decrease. After exposure at 6 °C for 72 h at WAF19, again *OeFAD2.2* and *OeFAD7* transiently rise, again with a quite similar trend in both genotypes, independently of the fact that, at this WAF, Canino is cold acclimated, whereas Moraiolo is not-acclimated. This means that *OeFAD2.2* and *OeFAD7* are *FAD* genes for cold response and not for acclimation in olive tree drupe. The same *FAD* genes, but also other *FADs*, may be active in other cultivars. It has been, in fact, reported that low temperatures increase the expression levels of *FAD6*, *FAD2-1*, and, mainly, *FAD2-2* in the mesocarp of Picual and Arbequina [45], and an RNAseq analysis has shown that *OeFAD2-2* increases its expression in response to cold also in the leaves of cv. Picual during early cold-exposure, being down-regulated under long-lasting cold exposure [46]. Taken together, independently of the differences among cultivars, it seems that *OeFAD2.2* is the prevalent cold-response *FAD*-gene in olive trees.

## 6. OeFAD2.2 Transcription and C18:2-Content Increase in the Seed Coat of the Cold-Acclimated Drupe, and the Endosperm Cutinisation Increases Accordingly

By an integrated transcriptional and lipid analysis in cv. Frantoio, it has been demonstrated that, even if differences in lipid reserve accumulation exist in the olive tree seed coat and embryo before (WAF14) and after (WAF22) the acquisition of cold acclimation by the drupe, a crosstalk between the seed compartments is present and involves a partitioning of unsaturated-FA enriched reserves [17].

The transcription of the *FAD* genes specifically involved in the drupe oleogenesis and cold response, i.e., the two isoforms of *OeFAD2*, and *OeFAD7* (previous paragraph), has been analyzed by D’Angeli and co-workers [17]. The transcript abundance of all the three *FADs* increases in the embryo at WAF22 in comparison with WAF14, but strongly only in the case of the two isoforms of *OeFAD2*. *OeFAD7* transcript abundance is ca. 1/80 and 1/17 of *OeFAD2.1* and *OeFAD2.2*, respectively, and, accordingly, C18:3 (i.e., the product of OeFAD7 activity) levels are quite undetectable. The increases in *OeFAD2* transcripts do not couple with an increase in C18:2 levels, which, by contrast, are reduced in all the fractions, i.e., free fatty acids (FAs), TAGs, and polar lipids (PLs), leaving C18:1 as the main unsaturated lipid reserve of the embryo.

**Figure 3:**
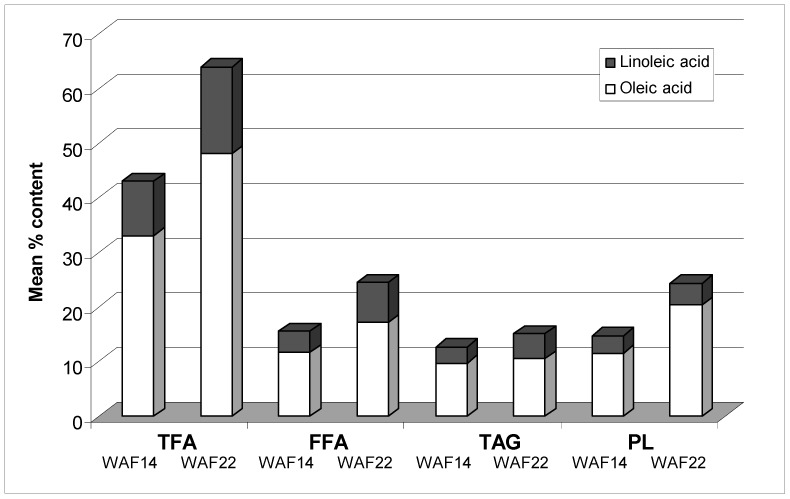
Comparison of C18-unsaturated fatty acids (FAs) present in the lipid fraction of seed coats from drupes of cv. Frantoio at 14 and 22 weeks after flowering (WAFs). The mean percentage content of oleic acid (C18:1) (white part of the column) and linoleic acid (C18:2) (black part of the column) in the total FA (TFA), free FA (FFA), triacylglycerol (TAG), and polar lipid (PL) fractions are shown. C18:3 was undetectable. Lipid classes were separated by thin layer chromatography (TLC) after quantitative extraction and dehydration according to D’Angeli et al. [17]. Spots corresponding to the different fractions were recovered from the plates, transmethylated by borontrifluoride, and the resulting FA-methyl esters analysed by GC-FID. Seed coat samples came from the same seed batches used in [17]. Data are from three unpublished independent determinations.

Interestingly, this is not the case of the seed coat. *OeFAD2.2* levels show a more than 70-fold rise at WAF22 in comparison with WAF14, whereas *OeFAD2.1* only a small rise, and *OeFAD7* transcription is always low. The expression of these genes result into strong increases in C18:2 levels (Figure 3), and into quite undetectable levels of C18:3. Oleic acid is the main unsaturated fatty acid present in the seed coat, and mainly in the free fatty acid (FFA) and polar lipid (PL) fractions (Figure 3). Linoleic acid shows the highest level in the FFA fraction (Figure 3). Both C18:1 and C18:2 are known components of seed cutin [47]. Their production seems necessary for the building up of endosperm cuticle, in accordance with the histological results (Figure 2G,H).

By cytosolic calcium signalling investigations, and over-expression and immuno-localization studies, the tobacco PR-5 protein osmotin has been proposed as a cryoprotectant for olive tree leaves, because its over-expression blocks cold-induced calcium-transients in non-cold-acclimated protoplasts [12]. An osmotin-like protein with cryoprotective activity has been also found in *Solanum dulcamara* [48]. Moreover, an osmotin involved in cuticle biogenesis is active in tomato fruit [49]. An *OeOSM* gene has been found in olive trees [7,17]. The immunolabelling technique reveals the absence of osmotin in the embryo, but the presence in the seed coat at WAF22 (cells of the inner epidermis and the outermost endosperm layers, Figure 2I). A possible role of osmotin as a lipid-transfer protein has been suggested for olive tree seeds. The lipid-transfer activity of the protein seems, in fact, essential for the cuticle formation of the endosperm. The production of free C18:1, and mainly C18:2 by OeFAD2.2, seems functional to cutin build-up in the seed coat inner epidermis. The exocytosis of the larger aggregates (Figure 2G,H), named cutinosomes [50], by the activity of osmotin, directed from the inner seed coat epidermis to the outer endosperm, increases endosperm cuticle, causing the protection of the non-cutinised embryo, thus allowing its desiccation tolerance.

## 7. OeFAD8 Expression Is a Determinant for Cold-Acclimation in Drupes and Leaves, and Is Associated with Parallel Changes in C18:3-Lipids and in Cutinisation

Olive trees maintain the leaves all year and, in the Mediterranean Basin, the drupes remain on the tree for months during fall/early winter. Transient increases in cytosolic calcium are among the early responses of both leaf and drupe protoplasts to cold stress, ceasing when cold acclimation is acquired [5,6,12]. This suggests that the two types of aerial organs are subjected to the same acclimation program to allow plant survival. The cuticle provides a hydrophobic protection for aerial plant organs, and shows dynamic qualitative and quantitative changes in response to the environment [28,51]. For example, in olive trees water deficit triggers an increase in cuticle thickening as an adaptation to summer drought [52]. Moreover, in numerous plants, the formation of a thicker cuticle also occurs as a protection to cold-induced winter drought [53]. Leaf and fruit cuticles generally contain the same classes of compounds, however, genotype-specific-changes are possible, as, in fact, occurs in olive trees in response to drought and pathogens [52,54]. In a previous paragraph, it has been shown that cutinosomes, enriched in C18:2-FA, are extruded by the seed coat to the endosperm to cause an indirect protection for desiccation to the non-cutinised embryo, with osmotin involved in this lipid-trafficking (Figure 2I). However, the embryo does not become cold acclimated [17]. It has been demonstrated that a differential remodelling of the lipidome during cold acclimation, involving changes in unsaturated TAGs, occurs in the natural accessions of *Arabidopsis thaliana* [55]. The activity of the unsaturated FADs is not only required for cutinisation, but also, and mainly, for membrane restructuring related to cold acclimation, because increased desaturation of glycerolipids serves to compensate for the cold-caused decrease in membrane fluidity [56]. However, in olive tree drupe, *OeFAD2.2* and *OeFAD7* expression is enhanced by cold, but unrelated to cold acclimation, with the expression of the two genes ceasing at the end of oleogenesis [5]. As also mentioned before, the same genes are active in the leaves of the cold-tolerant cv. Picual during early cold-exposure, but are down-regulated during long-lasting cold-exposure [46]. Moreover, increases in C18:3/C18:2 ratio in leaves and fruits are needed for enhancing cold tolerance [57]. This means that the activity of a C18:3-forming FAD is also necessary for olive trees. How olive trees decode the low temperature message into transcriptional and lipid changes leading to leaf and drupe acclimation needs investigation, however a possible role of an *OeFAD8* gene in causing cold acclimation in drupes and leaves has been recently proposed [7].

In the drupes of cv. Canino, *OeFAD8* transcripts highly increased between WAF12 and WAF16, whereas the remained low in the cold-sensitive cv. Moraiolo, and, at WAF19 (i.e., the acclimation time for Canino), OeFAD8 activity resulted into enhanced C18:3 levels. In fact, the total content of FAs was more than three-fold higher than in Moraiolo, and the quantity of C18:3 was 0.85% of the total FAs, whereas it was only 0.31% in Moraiolo. The percentage distribution of C18:3 on the total FAs was 0.5% in the TAG fraction, 0.23% in the PL fraction, and 0.12% in the FFA fraction, whereas it was 0.06%, 0.19%, and 0.06%, respectively, in Moraiolo. Thus, the C18:3-enriched TAG fraction was the prevalent one in Canino drupes, whereas the C18:3-PL fraction was the highest one in Moraiolo. The same trend occurred in the leaves, with a many-fold increase in *OeFAD8* expression at WAF19 in Canino, and a decrease in Moraiolo, and a higher C18:3 level in the former genotype in comparison with the latter. However, there is a difference between the two types of aerial organs in cv. Canino, because the leaves show a C18:3-content about four-fold higher than the drupes, and C18:3 is mainly present in the PLs and not in the TAGs. However, independently of this difference, a very thick cuticle is present in the external cell walls of both the drupe epicarp (Figure 2J) and the adaxial leaf epidermis (Figure 2K), with values of cuticle thickness many-fold higher than in the corresponding organs of Moraiolo.

This increased cutinisation positively couples with *OeOSM* expression, and the immunolocalization signal of the osmotin protein in both organs (Figure 2L,M). This increased cutinisation may be interpreted as an adaptation of Canino to strongly limit dehydration during winter by enhancing the resistance to water loss. The exocitosis of OBs is required for cutin formation [58], and occurs in the leaf epidermis and drupe epicarp as a part of the cold acclimation program, also needing the activity of osmotin to favour the apoplastic translocation of cutinosomes [7]. The C18:3-enriched TAGs in the OBs of the olive tree acclimating drupes may participate to cutinisation because it is involved in the formation of hydroxyl-FAs, as occurs in *Arabidopsis* [59]. Conversely, in Moraiolo drupes, cold-sensitive and fated to fall rapidly, there is a limited need (and presence) of C18:3-unsaturates, and the prevalent presence of C18:3 in the PL fraction may be interpreted as a cold-short-term membrane response, as observed in other plants [60]. Of course, even if at lower levels than in Moraiolo drupes, the C18:3-PLs are important also for Canino drupes to allow the membrane fluidity necessary for a long-term membrane cold-adaptation.

## 8. Concluding Remarks

**Figure 4:**
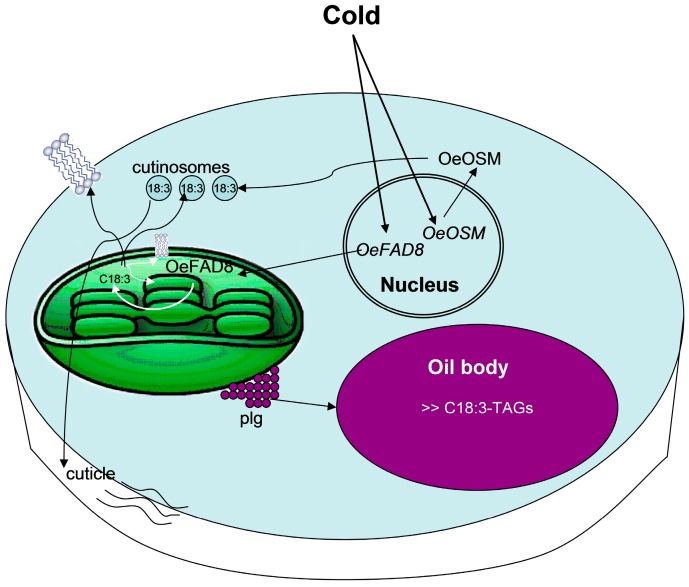
Model of the cellular mechanisms of the cold-acclimation response of olive tree drupe mediated by the orchestrated activities of OeFAD8 and OeOSM. Our hypothesis is that cold activates the transcription of *OeFAD8* and *OeOSM* in the nucleus. The formation of OeFAD8 occurs in the chloroplast, and causes the synthesis of C18:3. A part of this acid is used as C18:3-PLs to increase the fluidity of plasma-membrane and of chloroplast membranes. Another, more consistent, part extrudes from the chloroplast within the plastoglobuli (plg) as C18:3-TAGs. The plastoglobuli are fated to coalesce with the oil body, increasing its size and unsaturated content. However, another part of C18:3 is needed for C18:3-enriched cutinosome formation. OeOSM acts as a lipid trafficking protein favouring the extrusion of cutinosomes into the cell wall to increase cuticle formation. The model is modified from [7].

The results here presented and discussed show that lipid changes govern organ- and genotype-specific cold-induced processes in the olive tree. They occur by orchestrated roles of numerous FADs, specific lipid-transfer proteins, lipid unsaturation, and cutinisation, mainly involving C18:3-lipids in the drupe and leaf, and C18:2-lipids in the seed. A model summarizing our hypothesis about these integrated activities in the cold acclimated drupe is proposed (Figure 4).

## Abbreviations

OBs	Oil bodies
FA	Fatty acid
FAD	FA-desaturase
WAFs	Weeks after flowering
TAGs	Triacylglycerols
C18:1	Oleic acid
C18:2	ω6 linoleic acid
C18:3	ω3-Linolenic acid
ER	Endoplasmic reticulum
FFA	Free fatty acid
PL	Polar lipid
